# Effects of *Saccharomyces cerevisiae* on Pancreatic Alpha and Beta Cells and Metabolic Profile in Broilers

**DOI:** 10.1007/s12602-024-10397-y

**Published:** 2024-11-16

**Authors:** Silvana J. Peña B., Johan S. Salazar J., Jhon F. Pardo, Maria L. Roa, José R. Corredor-Matus, Julieta E. Ochoa-Amaya

**Affiliations:** 1 Universidad de los Llanos, Villavicencio, Colombia; 2 Facultad de Ciencias Agropecuarias y Recursos Naturales, Universidad de los Llanos, Villavicencio, Colombia; 3 Research Group On Pathology of Domestic and Wild Animals, Universidad de los Llanos, Villavicencio, Colombia

**Keywords:** Blood chemistry, Lipid profile, Yeast, Insulin-secreting cells, Glucagon-secreting cells, Pancreas (sources: DeSC, CAB)

## Abstract

To evaluate the impact of *Saccharomyces cerevisiae* (SC) supplementation on pancreatic islet areas, alpha and beta cell populations, blood glucose levels, and lipid profiles in broilers, broilers were randomly assigned to two groups: a control group (T1) without SC and a treatment group (T2) supplemented with SC. Islet areas, alpha and beta cell counts, serum glucose and insulin levels, and lipid profiles were assessed. SC supplementation significantly decreased blood glucose levels compared to the control group. Additionally, HDL cholesterol levels were elevated in the SC-supplemented group. Although insulin levels remained unchanged, SC supplementation altered the correlation between pancreatic islet areas and alpha and beta cell populations, suggesting a potential influence on pancreatic islet function. Dietary supplementation with *Saccharomyces cerevisiae* can improve glycemic control and lipid profile in broilers. These findings highlight the potential benefits of using SC as a dietary additive in broiler production.

## Introduction

Today’s poultry farming faces great challenges as regards nutrition due to increasing competition for the cost of commodities used in animal and human nutrition, calling for alternative ways to feed farm animals. Furthermore, to achieve higher weight gains, antibiotics have been overused as growth promoters, with the undesired side effect of increasing bacterial resistance. Hence, novel products, such as probiotics, have recently been proposed as feed supplements since they can be safely employed in animal nutrition and be highly beneficial to both animals and consumers [1].

*Saccharomyces cerevisiae* (SC), a yeast belonging to the probiotic family, is considered a promising alternative to growth-promoting antibiotics (GPA) [1]. In previous works published by the group, the effects on the digestive tract have been investigated, and among the histomorphological findings found are that the villi present in the duodenum of broilers supplemented with probiotics had a greater area (*p* = 0.0127), a greater basal width (*p* = 0.0049), and a greater apical width (*p* = 0.0024), as well as a greater crypt area (*p* = 0.0189). Significantly higher levels of mucus were noted in the duodenum (*p* = 0.0480) and jejunum (*p* = 0.0480) of broilers supplemented with probiotics [1]. A similar study found that the group supplemented with SC presented a greater area of the crypts in the duodenum (*p* = 0.0119) and jejunum (*p* = 0.0355), a smaller number of crypts per millimeter in the duodenum (*p* = 0.0420), and higher mucus production in the duodenum compared to the control group (*p* = 0.0185), while in the jejunum, no significant differences were observed [2]. However, the effects of SC supplementation on the physiology of broilers are assessed by changes in blood biochemistry, but the correlation between those changes and histological findings is not known yet.

Therefore, this study aimed to assess the effects of supplementation with the probiotic SC on blood chemistry parameters (triglycerides, total cholesterol, HDL, LDL, glucose, and insulin) in broilers and correlate them with histological findings in pancreas tissues (areas of islets of Langerhans and *α*- and *β*-cells), liver, central splenic and coronary arteriole tissues, and glucose and insulin levels.

## Materials and Methods

### Location

The study was conducted at the Barcelona campus of University of Llanos, in the city of Villavicencio, Department of Meta, Colombia, at an altitude of 420 m above sea level, average temperature of 28 °C, annual rainfall of 4.050 mm, and relative humidity of 85%. The blood parameters of the broilers under investigation were analyzed at Animal Labs located in the city of Villavicencio.

### Experimental Design

Tissues obtained from 45-day-old Cobb500 chickens were used. The individuals belonging to the study were raised in the same shed and divided into cages, considering the control and SC-supplemented groups. The individuals had water and food ad libitum; they were fed with a commercial starter diet during the first 15 days according to the manufacturer’s instructions and fattening; both the control group and the SC-supplemented group had similar food consumption. Supplementation with the probiotic began on day 15 with 5 days of habituation, starting records on day 20 of its consumption. The probiotic used corresponded to a commercial product; 5 mg of the dry product was administered for each kilogram of the commercial diet, ensuring the concentration of 10^7^ CFU/g of the probiotic in the experimental diet. The records were taken from day 20 of the individuals’ lives until the end of the experiment on day 45 of the individuals’ lives. The entire study was carried out at the Barcelona farm of the University of Llanos.

The project was developed with the prior approval of the Bioethics Committee of the University of Llanos, meeting in July 2021, which authorized the use of histological tissues deposited in the histopathology laboratory. The samples were obtained from broilers treated with the probiotic SC. Twenty-four animals were selected from a batch of 200 broilers admitted to Barcelona farm. They were divided into two treatment groups: T1 (control, without probiotics; *n* = 10) and T2 (with SC; *n* = 14). Blood samples were collected in red cap vacutainer tubes and taken to the Physiology Laboratory at the University of Llanos where their sera were separated by centrifugation and stored at − 20 °C. Triglycerides, total, HDL, and LDL cholesterol, and glucose levels were measured by spectrophotometry on an automated A15 BioSystems Clinical Chemistry equipment. Insulin levels were determined by ELISA tests using a 96-well Monobind Insulin AccuBind ELISA kit on a Multiskan Ex Thermo Lab Systems ELISA reader.

The histological investigation employed waxed blocks of pancreas tissues from SC-supplemented broilers tended by the institutional project “Uso de harina de Cayeno Hibiscus rosa sinencis y Cajeto trichanthera gigantes y Gliricidia sepium más probiótico *Saccharomyces cerevisae*, sobre los parámetros productivos y de digestibilidad en pollos de engorde.” Two treatments were used: T1 (control; without SC) and T2 (supplemented with SC at a concentration of 10^7^ cfu/g in the experimental diet). Tissues were processed according to histotechnical protocols at the histopathology laboratory of the University of Llanos. For paraffinization, they were subsequently cut up and stained with hematoxylin–eosin.

For the analysis of the splenic arteries, 5 histological fields with splenic arteries were selected for each individual, and photos were taken using a microscope (Leica DM 500) and a LEICA ICC500 camera using LAS EZ software at 10x and 40x magnifications. Using ImageJ software, the fat vacuoles between the tunica intima and the tunica adventitia of the splenic arteries were counted (40x).

Glycogen content in the liver was determined by measuring the colored areas (%) using PAS staining, according to the methodology described by Martínez-Sánchez et al. [3]. Photographs taken of the micropreparations at 10x were used to quantify the area according to the colors presented (PAS positive magenta) giving the percentage of glycogen/field/animal. For measurements of PAS-positive areas, ImagePro Plus 5.0 software (Media Cybernetics, Silver Spring, MD, USA) was used.

Areas of islets of Langerhans were determined by photomicrography (40x) and the ImageJ 1.52b software. Islet areas were measured according to the method described by Cruz-Ochoa et al. [4]. Five islets were established in the tissue samples per animal (*n* = 5). On the other hand, areas of *α*- and *β*-positive cells were determined by immunohistochemistry for each islet of Langerhans (40x) for both treatments, using the ImageJ 1.52b software. Immunohistochemical assessment was done by determining positive areas through the modified grade process; five fields were observed in each islet, five islets per slide.

Immunohistochemical analysis (IHC) of pancreas tissues employed 5 µ tissue sections, deparaffinized, and rehydrated by means of baths in solutions with decreasing gradients of ethanol concentration. It employed the anti-glucagon primary antibody (clone EP74) and the Master polymer plus detection system (peroxidase), including DAB chromogen, respectively. Antibodies for insulin clone EP125 presentation 7 ml (INSULIN MAD-0007QD) and Master polymerplus detection system (peroxidase) (includes chromogen) 10 ml (MAD-00237QK-10).

### Statistical Analysis

Excel databases were created, and a parametric statistical analysis was conducted. The D’Agostino and Pearson omnibus and normality test was performed. All the data showed a normal distribution. The probiotic-supplemented (T2) and control (T1) groups were analyzed using a “t” test for unpaired samples. All statistical procedures were conducted with the GraphPad Instat software, version 5.01 (1992–2007), with *p* < 0.05. Additionally, the RStudio software was used to perform Pearson correlations between the blood chemistry data obtained through the investigation of histological parameters in the pancreas, coronary and splenic artery, and liver tissues from T2 animals and those from T1 animals.

The objective of this study was to assess the effect of supplementation with SC on areas of islets of Langerhans and alpha and beta cells (*α*-*β*-cells) in broilers by immunohistochemistry (IHC) and to correlate areas of *α*- and *β*-cells in relation to pancreatic islet area, glucose, and insulin in both SC-supplemented and control groups. To correlate data obtained for triglycerides, total, HDL, and LDL cholesterol, and histological parameters of vacuolar assessment in central splenic arteriole and coronary arteries in both treatments.

## Results

The unpaired “t” test revealed statistically significant differences (*p* < 0.05) in the blood chemistry of SC-treated broilers (Table 1), such as higher HDL cholesterol levels (*p* = 0.0365) and a drop in glucose levels (*p* = 0.0118). No statistically significant differences were found in insulin, total and LDL cholesterol, and triglycerides despite their levels exhibiting a slight downward trend.

**Table 1:** Effects of SC supplementation on total, HDL, and LDL cholesterol, triglycerides, glucose, and insulin in control and probiotic-treated broiler sera (*n* = 24)

Parameter	Groups	
	**Control (T1)**	**SC-treated (T2)**
Total cholesterol (mg/dL)	153.9±18.41	124.0±5.97
HDL cholesterol (mg/dL)	12.20^a^±0.8424	14.54^b^±0.979
LDL cholesterol (mg/dL)	111.3±15.83	86.15±5.164
Triglycerides (mg/dL)	135.3±14.16	111.7±6.191
Glucose (mg/dL)	297.9^a^±21.18	244.7^b^ ±7.5
Insulin (ng/mL)	0.5433±0.09	0.8861±0.35

^ab^Means whose index does not have any letters in common are statistically different. Note: data are expressed as means ± standard error; unpaired “t” test, *p* < 0.05

**Fig. 1:**
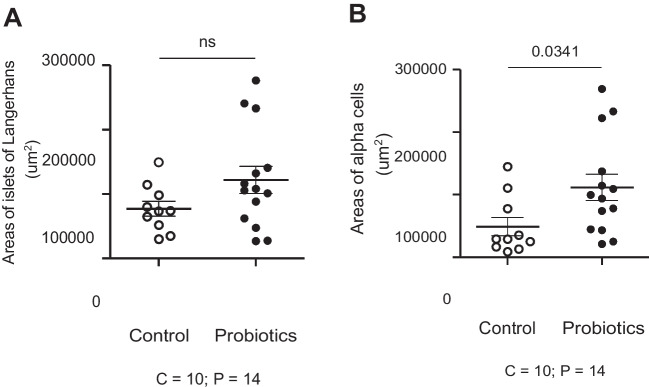
Effect of probiotic supplementation on areas of islets of Langerhans and glucagon-positive α-cells

**Fig. 2:**
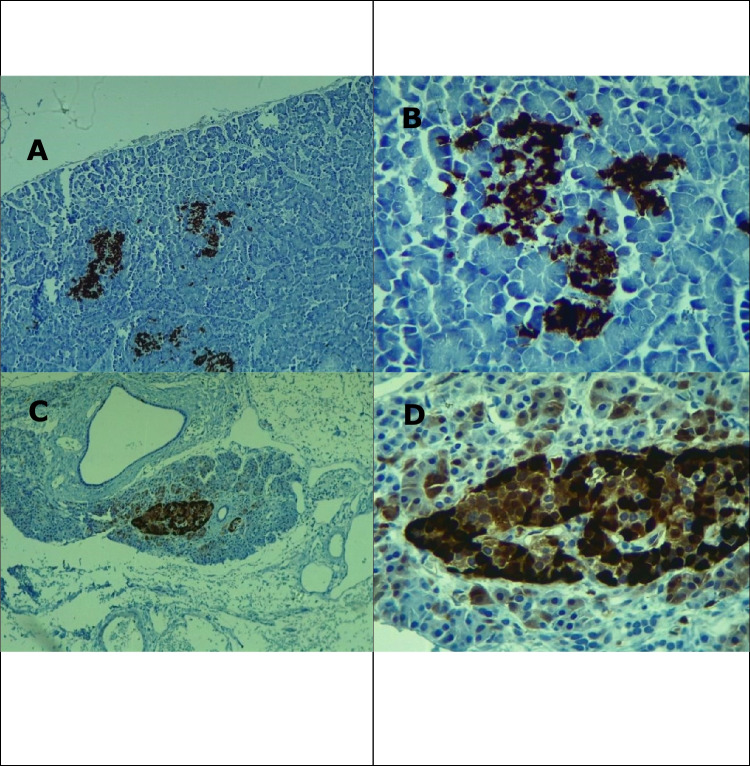
Microphotography of areas of islets of Langerhans in control group (**A** = 10x; **B** = 40x) and probiotic-supplemented group (**C** = 10x; **D** = 40x). Immunohistochemical analysis (IHC) for glucagon *α*-cells. Source: Salazar (2022)

The unpaired “t” test showed no statistical differences (*p* > 0.05) in the probiotic-treated group (121,872 ± 20,941) µ^2^ and control group (77,033 ± 11,486) µ^2^ in relation to the area of islets of Langerhans (*p* = 0.1076). Notwithstanding, statistical differences (*p* = 0.0341) were found in relation to the alpha cells in islets of Langerhans in the probiotic-supplemented group (111,850 ± 20,995) µ^2^ and control group (48,965 ± 14,620) µ^2^ (Figs. 1A, B and 2A-D).

Figure 2A–D shows the positive area for glucagon-producing α-cells in T1 (control group) and T2 (SC-supplemented group).

**Fig. 3:**
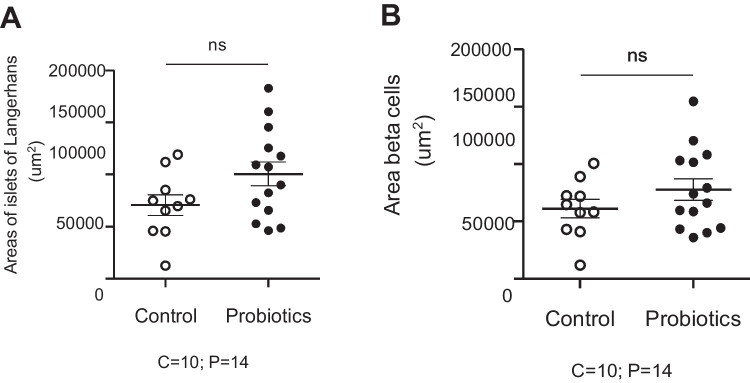
Effect of probiotic supplementation on areas of islets of Langerhans and insulin-positive cells

**Fig. 4:**
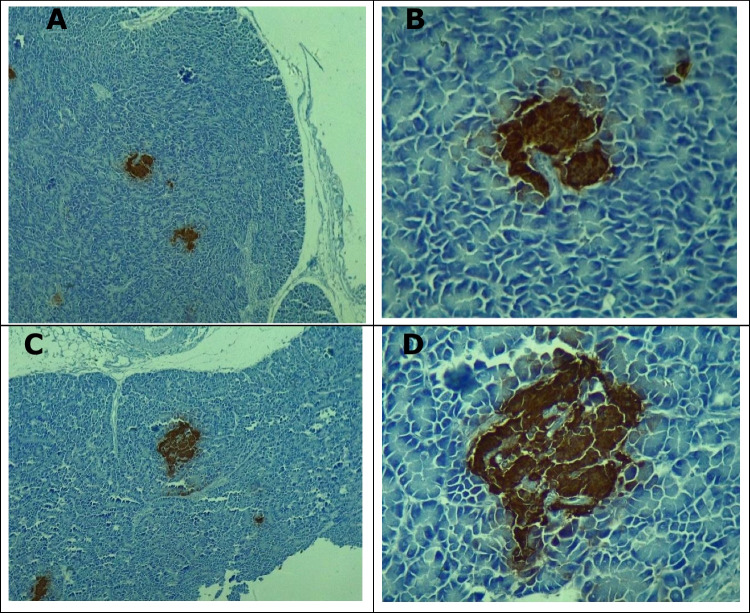
Microphotography of areas of islets of Langerhans in the control group (**A** = 10x; **B** = 40x) and in the probiotic-supplemented group (**C** = 10x; **D** = 40x). Immunohistochemical analysis (IHC) for insulin *β*-cells. Source: Pardo (2022)

The unpaired “t” test revealed no statistical differences (*p* > 0.05) in T2 (92.440 ± 11.360) µ^2^ and T1 (70.540 ± 9.996) µ^2^ in relation to the area of islets of Langerhans (*p* = 0.1955). Likewise, no statistical differences (*p* = 0.4188) were found for *β*-cells in islets of Langerhans in T2 (71.950 ± 9.105) µ^2^ or T1 (61.110 ± 8.001) µ^2^ (Figs. 3A, B and 4A-D).

Figure 4A–D shows the positive area for insulin-producing β-cells in T1 (control group) and T2 (SC-supplemented group).

### Correlations

Table 2 points to significant differences (*p* < 0.05) found for T1 (control group; without SC supplementation) when blood parameters (total cholesterol, HDL cholesterol, LDL cholesterol, VLDL cholesterol, triglycerides, glucose, and insulin) were correlated with histological features in the pancreas (40x area for *α*- and *β*-cells in islets of Langerhans), coronary and splenic arteries (fat vacuole count), and liver (percentage of glycogen, infiltrated area, PAS + area, zone III inflammatory infiltration, and zone III necrosis).

**Table 2:** Treatment 1 correlations between measured blood chemistry parameters and histological variables in the pancreas, liver, and coronary and splenic artery tissues

Parameter 1	Parameter 2	*r*	*p*-value
Glucose	Triglycerides	0.91	0.024*
Total cholesterol	LDL cholesterol	0.98	< 0.001***
Total cholesterol	Triglycerides	0.93	0.014*
Langerhans islet area (40x)	*α*-cell area	0.85	0.0033**
Langerhans islet area (40x)	*β*-cell area	0.98	< 0.0001***
% glycogen	PAS + area liver	0.98	< 0.001***
*α*-cell area	Serum glucose	0.49	0.1825
*β*-cell area	Serum insulin	− 0.36	0.15

*p< 0.05, **p< 0.01, ***p< 0.001

No statistically significant correlations were found between blood parameters and histological data for coronary and splenic arteries (fat vacuole count).

In T1, glucose and triglycerides exhibited a highly significant positive correlation (*r* = 1). Triglycerides are the major constituent of the low-density lipoprotein (VLDL) fraction with a high and significant correlation (*r* = 0.93), whereas LDL cholesterol is the main lipid associated with total cholesterol (*r* = 0.98). This implies that rising glucose promotes an increase in triglycerides and LDL and total cholesterol levels.

Histological investigation in T1 found a positive and significant correlation between the area of islets of Langerhans and the areas of *α*- and *β*-cells in the islets (*r* = 0.85 and 0.98, respectively). In the liver, a positive and highly significant correlation (*r* = 0.98) was found between the percentage of glycogen and the area of PAS + . No significant differences (*p* > 0.05) were found when *α*-cell data were correlated with serum glucose levels for this group, nor between serum insulin levels and *β*-cell area, despite their correlation being slightly negative (*r* = − 0.36).

Table 3 shows correlations for the SC-supplemented group (T2), with significant differences (*p* < 0.05) when blood parameter results (total cholesterol, HDL cholesterol, LDL cholesterol, VLDL cholesterol, triglycerides, glucose, and insulin) were correlated with histological variables in pancreas (40x area; cell count in islets of Langerhans), liver, and coronary and splenic artery tissues (fat vacuole count).

**Table 3:** Treatment 2 correlations between blood chemistry parameters and histological variables in the pancreas, liver, and coronary and splenic artery tissues

Parameter 1	Parameter 2	*r*	*p*-value
Glucose	Insulin	− 0.84	0.009**
Total cholesterol	LDL cholesterol	0.80	0.035*
Islet area (40x)	*α*-cell area	0.99	< 0.0001***
Islet area (40x)	*β*-cell area	0.92	< 0.0001***
*α*-cell area	*β*-cell area	0.78	0.0003***
% glycogen	PAS + area liver	0.98	< 0.001***
*α*-cell area	Serum glucose	− 0.47	0.07
*β*-cell area	Serum insulin	0.04	0.44

*p< 0.05, **p< 0.01, ***p< 0.001

Finally, probiotic supplementation was observed to decrease serum glucose in T2 broilers (SC-supplemented animals), which caused blood insulin levels to vary with a negative and significant correlation (r = − 0.84). No statistically significant correlations were found in T2 between blood parameters for serum glucose and histological findings for α-cells in islets of Langerhans.

In the SC-supplemented group (T2), a significant positive correlation was observed between total cholesterol and its LDL fraction. Similarly, T2 histological findings in pancreas tissues (area of islets of Langerhans; 40x) exhibited a significant positive correlation (*r* = 0.99) with the *α*- and *β*-cell count in islets of Langerhans (*r* = 0, 92). Also, a moderate (*r* = 0.78) and highly significant (*p* = 0.0003) correlation was observed between *α*- and *β*-cells in islets of Langerhans for T2 broilers.

Likewise, the findings in liver tissues showed a positive and highly significant correlation (*r* = 0.98) between glycogen percentage and PAS + area. It is possible to observe the effect of yeast supplementation on the area of *β*-cells and level of serum insulin; the correlation between these parameters was positive, albeit with non-significant (*p* = 0.44) and slight (*r* = 0.04). That is, the yeast enlarged the area of *β*-cells in T2, which caused the serum insulin level to rise.

**Fig. 5:**
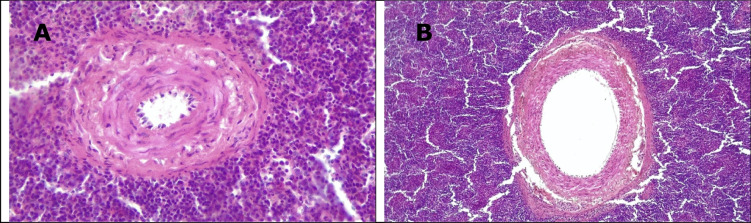
**A** Microphotography of spleen H&E 10x, rounded and elongated translucent vacuoles are observed in the muscular layer of a splenic arteriole from the control group. **B** Spleen H&E 10x, vacuolar degeneration is observed in the middle layer of a splenic arteriole from the group supplemented with *Saccharomyces cerevisiae*. Source: Rodríguez (2020)

There is evidence of a difference in the total number of vacuoles of central arterioles of the splenic germinal centers caused by the supplementation of *S. cerevisiae* with respect to its controls (Fig. 5A, B).

Figure 5A, B shows the histological sections of the spleen from the control group (Fig. 5A) and from the group supplemented with probiotic *Saccharomyces cerevisiae* (Fig. 5B; hematoxylin and eosin staining (H&E)).

## Discussion

In this study, the broilers supplemented with the probiotic SC exhibited a significant drop in glucose levels, a finding that coincides with that reported in the literature. Was reported a significant decrease in serum glucose after supplementing groups of rabbits with SC in doses of 4 and 10 g/kg [5] and was reported by [6] a glucose reduction at the 14–42 days in SC-treated piglets. Likewise, found a significant drop in glucose levels (*p* < 0.05) in SC-supplemented broilers (1.5 g/kg) [7] and [8] reported a decrease in serum glucose levels in broilers fed rubber tree seeds plus 1% SC and 1% SC.

A significant negative correlation between serum glucose and insulin was found in T2. The avian pancreas has been described as containing minute amounts of insulin, i.e., it has low insulinogenic potential, whose release is slow in response to glucose load. Insulin release in birds is unaffected by fasting, hormones, or vagal stimulation. In in vitro avian systems, insulin has no antilipolytic effect and is not very lipogenic, different from mammals and humans [9].

SC has mannooligosaccharides (MOS) on the surface of its cell walls and mannose structures that increase the expression of the MYH7 gene at the intestinal level. MYH7 encodes a β-isoform of the myosin heavy chain, which is responsible for muscle movement and active passage of glucose from the intestinal lumen into the blood. SC also stimulates the expression of NPPA, the precursor to the atrial natriuretic factor. NPPA is a vasodilator that improves blood flow, a mechanism that controls the passage of glucose from the intestinal lumen into the portal vein, thus increasing blood glucose [10]. And cultures can facilitate food digestion as they contain digestive enzymes, such as amylase, maltase, sucrase, lactate dehydrogenase, proteinase, polypeptides, dipeptidase, deaminase, transaminase, lipase, phospholipase, phosphatase, and phytase. In the absence of these enzymes, sugars cannot be fermented by SC to produce carbon dioxide and ethyl alcohol [11].

Consumption of probiotics may be associated with lower blood glucose and cholesterol levels, immune system stimulation, and other benefits due to their surface polysaccharides and short-chain fatty acid metabolites [12]. In the SC-supplemented group (T2), glycemia levels decreased without altering the area of *β*-cells as the amount of insulin-regulating glycemia in this group is lower and, therefore, basal glucose levels are needed for the survival of *β*-cells in pancreatic islets [13]. It is possible to affirm that a decrease in glycemia levels in SC-supplemented broilers caused the area of *α*-cells to expand significantly to maintain glycemia at constant levels. Despite the correlations between glycemia and *α*-cell area not being significant, probably because the probiotic promoted glycemia homeostasis in T2, a relationship can be observed, indicating communication between *β*-cells and *α*-cells [14].

The control treatment group (T1) exhibited a highly significant positive correlation between glucose and triglycerides. Today’s constant pressure to improve productivity in animal production at poultry farms can affect intestinal homeostasis by altering the intestinal microbiota of commercial birds [15]. This, together with excessive use of fatty acids and carbohydrates, can generate energy imbalance in diets high in carbohydrates and low in protein [16], like what happens in human metabolic diseases [17]. This imbalance affects the absorption of lipids, interfering with the conversion of bile salts [9] and increasing LDL, total cholesterol, and triacylglycerols.

As to the correlations of histological findings in pancreas tissues, the area of islets of Langerhans (40x) is closely related to *α*- and *β*-cell areas in the islets for both the control and SC-supplemented groups. As glucose regulates the survival and proliferation of *β*-cells in a dose-dependent manner, varying its load could be partly responsible for reducing or increasing the volume of pancreatic islet cells [18].

Beta cells are known to fulfill the function of expressing, processing, and secreting insulin in proportion to continuously varying concentrations of circulating nutrients [16]. Islets of Langerhans, in turn, constitute around 1.5% of the volume of the organ [19]. High glucose levels may have beneficial effects, e.g., stimulation of *β*-cell proliferation [11] since glucose is a natural mitogen for *β*-cell replication in early hyperglycemic episodes [9]. In this study, this was not observed in the SC-supplemented group (T2) since the area of *β*-cells did not expand, probably due to hypoglycemia caused by the probiotic in question. There was an increase in the area of *α*-cells, which were able to fulfill the functions of *β*-cells [20].

The correlation between the areas of *α*-cells and *β*-cells in islets of Langerhans was moderate (*r* = 0.78) and very significant (*p* = 0.0003), as shown in Table 3. This suggests a physiological and bidirectional synchronous interaction between both cell populations to maintain glycemia homeostasis in T2 broilers. As the area of *α*-cells changes due to the effect of the probiotic yeast, so does the area of *β*-cells.

Evidence was found for a significant increase in the area of *α*-cells in the SC-supplemented group (T2) as compared to the control group (T1). It should be noted that due to the high immunohistochemical staining of the plates corresponding to the experimental groups, it was not possible to count the nuclei to verify whether this result was due to an increase in the area of *α*-cells (hypertrophy) or in the number of *α*-cells (hyperplasia). However, a synchronous population can be inferred from the correlations obtained, corroborated by some authors [21].

Moreover, studies conducted on blood microcirculation point to important intercellular paracrine communication between *α*-cells and *β*-cells [22]. In addition, there should be considered studies showing the functional capacity of *α*-cells to replicate and fulfill the functions of *β*-cells by means of self-ablation [18]. These new approaches and studies regard the epigenetic regulation of islet DNA methylation as a regulatory mechanism in the differentiation and maturation of pancreatic *α*- and *β*-cells [23].

Contrary to that found in the control group (T1), the probiotic-treated group (T2) exhibited a positive and significant correlation between *β*-cells and islets of Langerhans. That is, the area of *β*-cells contributed greatly to the total area of islets of Langerhans. Moreover, a significant decrease in glucose was observed in T2 (244.7 ± 7.5; *p* = 0.0118) as compared to T1 (297.9 ± 21.18), probably because of supplementation with SC on serum insulin: it inhibited peaks or variations in it.

Similarly, in the SC-supplemented group (T2), serum insulin exhibited a slight increase (0.8861 ± 0.35), not significant in relation to T1 (0.5433 ± 0.09) but higher than the reference value in broilers (0.362–0.842 ng/ml [7]. Similar results were reported, which suggest that probiotic supplementation can increase insulin secretion from pancreatic *β*-cells by stimulating the homeobox-1 factor (PDX-1). On the other hand, maintained that the pancreas of birds contains small amounts of insulin, i.e., it has little insulinogenic potential, whose release is slow in response to high glucose load [7], which was not observed in T2.

The ability of β-cells to release insulin in response to glucose in the blood is due to their unique metabolism [17]. Beta cells constitute the only source of circulating insulin, and a loss of modulated insulin secretion can lead to dysregulated energy homeostasis [24]. In turn, the insulin secretory response is finely tuned to extracellular signals and intrinsic properties of *β*-cells [25]. Therefore, it should be recognized that nutrient sensing by *β*-cells occurs mainly through modulated glucose metabolism, which enables tight coupling between circulating glucose levels and insulin release [12].

In addition, glucose homeostasis is sustained by means of interdependent maintenance of insulin action in peripheral tissues, e.g., liver and muscles, along with basal and nutrient-dependent modulation of insulin secretion of *β*-cells in islets of Langerhans [26]. In liver tissues, a strong positive correlation was found between glycogen percentage and PAS + area. Hepatocytes produce the enzymes needed for gluconeogenesis, i.e., conversion of pyruvate into glucose [27].

Physiologically, glucagon is released in response to different metabolic signals, such as changes in glycemia. Increased plasma glucagon leads to an increase in hepatic glucose production in situations of hypoglycemia, counteracting the hypoglycemic effects of insulin.

Several researchers have proposed the complete mechanism of glycemia control at the metabolic level [28, 29]. According to these authors, when glucagon binds to its transmembrane receptor, conformational changes are generated to activate G proteins, increasing cAMP levels and activating the enzyme adenyl cyclase. This, in turn, activates the protein kinase A (PKA) and cAMP-response element binding protein (CREB).

CREB promotes the transcription of glucose-6 phosphate and phosphoenolpyruvate carboxykinase (PEPCK), which stimulates gluconeogenesis. At the same time, activated PKA generates intracellular changes by phosphorylating CREB, leading to the activation of different enzymes that increase fructose 6-phosphate and induce gluconeogenesis, while reducing the levels of fructose 2,6 bisphosphate and, consequently, glycolysis. On the other hand, many PKA-induced reactions cause pyruvate levels to drop, thereby suppressing glycolysis and activating phosphorylase kinase, which, in turn, contributes to a decrease in the levels of glycogen and hepatic glucose release.

In this study, SC-treated broilers (T2) exhibited a significant increase in HDL cholesterol, consonant with other authors that found an increase in HDL cholesterol at 42 days of age, which suggests that these specimens had better metabolic functioning [4, 30] administered dry yeast of the Saccharomycetales order (*Yarrowia lipolytica*, YL) to turkeys in two doses, namely, 3% (YL3) and 6% (YL6), in feed mixtures and found that HDL cholesterol increased significantly in the YL6 group. Increased HDL cholesterol levels suggest that probiotics can contribute to the regulation of serum cholesterol concentrations and improve the lipid profile, a noteworthy option for optimal management of cholesterol levels in animals and humans; however, it requires further studies in order to clarify other effects and its applicability in the production and clinical field.

Formed in the intestine and liver, HDL lipoproteins contain cholesterol, proteins, and phospholipids with very few triglycerides [31]. Bacteria from probiotics fulfill their hypocholesterolemic function through cholic and deoxycholic acids, which are conjugated with glycine and taurine, respectively. These compounds enter the small intestine, are absorbed by it, and reach the liver. During absorption, conjugated acids are exposed to intestinal microflora and hydrolyzed by bacteria found in fermented food.

Several mechanisms have been proposed to explain the cholesterol-mediating effect of probiotics: [2] cholesterol uptake by probiotics, [32] production of the enzyme bile salt hydrolase (BSH) by probiotics, increasing excretion of fecal bile acids, [5] conversion of cholesterol to coprostanol through cholesterol reductase, promoted by probiotics, and [6] probiotic inhibition of reductase of hydroxymethylglutaryl coenzyme A (HMG CoA), an enzyme participating in the synthesis of cholesterol [33].

The effect of supplementation with 500 and 1000 mg/kg of live yeast (LY) versus the use of the antibiotic chlortetracycline (CTR) (75 mg/kg) on the metabolic profile of broilers was evaluated [34]. They found lower HDL levels (*p* < 0.05) in the LY-supplemented groups as compared to those in the CTR-treated group on days 21 and 42. Their study hypothesized that live yeast can inhibit cholesterol oxidation, resulting in less lipid deposition in blood vessels and, thus, promoting anti-cholesterolemic effects. However, this mechanism requires further investigation. As regards the positive relationship between total cholesterol and its LDL fraction, researchers have proposed it to be due to an increase in proteins that contribute to apoprotein production. Found a significant rise in HDL cholesterol, which can lead to a decrease in the LDL fraction and total cholesterol [35].

Probiotic supplementation with SC decreased glycemia without affecting the *β*-cell area in islets of Langerhans in the experimental group (T2). Supplementation with SC affected islets of Langerhans in T2, significantly increasing α-cell area. The group of broilers supplemented with SC exhibited stable serum insulin levels. The absence of insulin peaks may prevent the onset of type 2 diabetes.

A higher presence of liver glycogen was evidenced in the SC-supplemented broilers, which the literature attributes to the existence of mannanoligosaccharides in the cell wall of this yeast. This fact promotes gene expression for glucose entry into cells, giving rise to higher concentrations of liver glycogen available for metabolism in animals.

In conclusion, in this study, dietary supplementation with SC decreased serum glycemia without affecting insulin. It modified the correlation of areas of pancreatic cell population in islets of Langerhans and enlarged areas of *α*-cells vs *β*-cells, thereby maintaining blood glucose homeostasis. In addition, SC increased levels of high-density cholesterol (HDL) in the blood.

## Data Availability

No datasets were generated or analysed during the current study.
